# Reproducing the Few-Shot Learning Capabilities of the Visual Ventral Pathway Using Vision Transformers and Neural Fields

**DOI:** 10.3390/brainsci15080882

**Published:** 2025-08-19

**Authors:** Jiayi Su, Lifeng Xing, Tao Li, Nan Xiang, Jiacheng Shi, Dequan Jin

**Affiliations:** 1 School of Mathematics and Information Science, Guangxi University, Nanning 530004, China; sujiayi@st.gxu.edu.cn (J.S.); 2406301052@st.gxu.edu.cn (L.X.); 2406301025@st.gxu.edu.cn (T.L.); 2306301044@st.gxu.edu.cn (N.X.); 2306301031@st.gxu.edu.cn (J.S.); 2 Center for Applied Mathematics of Guangxi, Guangxi University, Nanning 530004, China

**Keywords:** few-shot learning, image detection, neural network

## Abstract

Background: Studies have shown that humans can rapidly learn the shape of new objects or adjust their behavior when encountering novel situations. Research on visual cognition in the brain further indicates that the ventral visual pathway plays a critical role in core object recognition. While existing studies often focus on microscopic simulations of individual neural structures, few adopt a holistic, system-level perspective, making it difficult to achieve robust few-shot learning capabilities. Method: Inspired by the mechanisms and processes of the ventral visual stream, this paper proposes a computational model with a macroscopic neural architecture for few-shot learning. We reproduce the feature extraction functions of V1 and V2 using a well-trained Vision Transformer (ViT) and model the neuronal activity in V4 and IT using two neural fields. By connecting these neurons based on Hebbian learning rules, the proposed model stores the feature and category information of the input samples during support training. Results: By employing a scale adaptation strategy, the proposed model emulates visual neural mechanisms, enables efficient learning, and outperforms state-of-the-art few-shot learning algorithms in comparative experiments on real-world image datasets, demonstrating human-like learning capabilities. Conclusion: Experimental results demonstrate that our ventral-stream-inspired machine-learning model achieves effective few-shot learning on real-world datasets.

## 1. Introduction

When humans encounter novel situations, the brain can quickly learn the features of new stimuli and adjust behavior accordingly. Inspired by the biological theories underlying this process in the human brain, researchers have proposed a series of brain-based models. These models are capable of achieving functions similar to those of the nervous system or its learning mechanisms. Among them, neural networks are currently one of the most popular brain-inspired models in machine learning. Neural networks consist of multiple interconnected neurons or units [1,2,3,4,5]. After years of development, the latest achievement in neural networks is known as deep learning. Compared to traditional neural networks, deep learning models typically have dozens to thousands of layers and exhibit more complex structures. With the emergence of efficient training methods, deep neural networks have demonstrated powerful learning capabilities and achieved remarkable results in image classification and related fields. Traditional machine learning methods require large amounts of labeled data for training, but in real-world scenarios, collecting such large-scale datasets can be extremely costly. However, neural networks based on backpropagation suffer from several limitations [6,7,8,9,10], such as high computational cost, poor model interpretability (the black-box problem), lack of biological plausibility, and strong dependence on large datasets [11]. These challenges have diminished their advantages when applied to few-shot learning tasks [12]. In daily life, humans learn and adapt to new things based on prior knowledge. Inspired by this, researchers have begun exploring whether knowledge acquired from core tasks can be transferred directly to related target tasks, thus overcoming the challenges neural networks face in few-shot learning [13,14].

For example, Peng et al. proposed the SgVA-CLIP method, which addresses the issue that vision-language pre-trained models may neglect detailed visual information when distinguishing between different images [15]. Bendou et al. introduced a method called EASY, which provides a clearer and more intuitive understanding to help people execute few-shot learning more effectively, while adding almost no hyperparameters or additional parameters to the training of initial deep learning models [16]. Zhang et al. proposed MetaQDA, a Bayesian meta-learning framework that extends the classic quadratic discriminant analysis (QDA) [17]. Hu et al. proposed the P>M>F method, a simple yet effective pipeline for few-shot learning [18]. Zhang et al. approached few-shot image classification from the perspective of optimal matching between image regions [19]. Rong et al. proposed ESPT (Episode-Based Self-Paced Training), which leverages a self-supervised learning framework to generate pre-training tasks for learning features without labels [20]. It helps the model better understand and utilize the structural information of images when facing new tasks, thereby enhancing its adaptability to new tasks and reducing performance degradation due to insufficient samples by incorporating this self-supervised pre-training task into few-shot learning tasks. Shalam et al. introduced the BPA method in 2024, aiming to enhance the feature representation of input items for downstream matching- or grouping-related tasks [21]. This method captures the complex relationships between features, provides a flexible and effective way to improve existing models, and achieves state-of-the-art results in various tasks. Fifty et al. proposed a method called CAML (Context-Aware Meta-Learning), which enhances adaptability to new tasks by incorporating contextual information [22]. CAML primarily addresses the issue of task diversity in meta-learning by introducing contextual information in task representation, effectively guiding the model to adapt to new tasks. Song et al. proposed a Transformer-based few-shot classification method that integrates multi-scale feature representations and a multi-scale matching strategy, significantly enhancing the model’s ability to understand objects of varying scales and improving classification performance [23].

Among the above-mentioned methods, some utilize unlabeled sample information to generate pseudo-labels or prototypes, thereby increasing the number of samples for model training. Others adopt transfer learning, applying knowledge obtained through pre-training on large datasets in the source task to the target task, thereby improving the few-shot learning of the model. These methods effectively enhance the performance of models that originally rely on large-scale samples for training in few-shot classification tasks. Furthermore, if we can develop a new model that is inherently suitable for few-shot learning, we may achieve even better few-shot image classification.

The learning process in humans requires the coordinated operation of the entire nervous system, whereas current neural network models can only achieve certain functions at a partial or microscopic level. Since humans are capable of few-shot learning, we aim to implement a more holistic, flexible, and multi-scale neural framework to realize few-shot learning mechanisms. Studies have shown that different brain regions respond differently to various input signals [24,25]. The brain determines the type of input signal based on the state of neural fields [26]. Inspired by this biological process, we attempt to design a few-shot learning model based on neural fields. This approach is interpretable from both biological and mathematical perspectives. In recent years, with advances in brain research technologies [27,28], we have gained a clearer understanding of the brain’s structure [29].

Inspired by the overall mechanism of the ventral visual stream [30,31,32], we designed a classifier for few-shot learning based on the neural field model. By coupling this classifier with the feature extraction part of the Vision Transformer (ViT) [4], we can compensate for ViT’s shortcomings in few-shot learning. Our model achieves experimental results that surpass those of the current state of the art on standard datasets while maintaining high efficiency and low cost.

## 2. Preliminaries

### 2.1. Few-Shot Learning

Few-shot learning aims to develop methods that can learn from a small number of labeled samples and generalize to a large number of unlabeled samples [33,34,35]. In few-shot learning, we typically use the N-way K-shot setting, where N denotes the number of classes and K represents the number of labeled images per class. Thus, a total of N × K labeled images are used during the training phase of few-shot learning.

### 2.2. The Ventral Visual Stream

**Figure 1:**
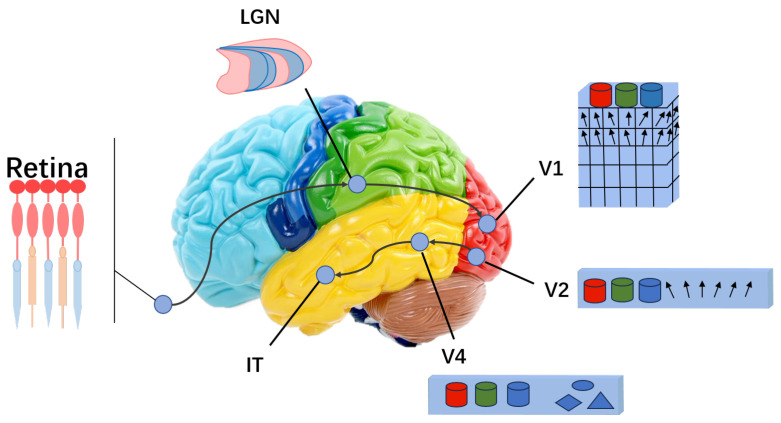
Part of the ventral stream structure, which is associated with human few-shot learning behavior. From a biological theoretical perspective, it has provided significant inspiration for our design of few-shot learning models and laid a solid biological theoretical foundation for the model proposed in this paper.

As shown in Figure 1, the part of the human brain that is associated with few-shot learning is the main pathway of the ventral visual stream. This pathway primarily includes the retina, lateral geniculate nucleus (LGN), and cortical regions V1, V2, V4, and IT. Based on their functional roles, we divide the main pathway of the ventral visual stream into two parts: the feature extraction part—comprising the retina, LGN, and cortical areas V1 and V2—and the memory and learning part, which includes cortical areas V4 and IT. Before simulating this pathway, let us first examine how it biologically accomplishes image recognition and learning.

As the starting point of the pathway, the retina is responsible for receiving external visual stimuli [36]. The cone cells and rod cells in the retina convert these stimuli into neural signals. Rod cells are sensitive to light and enable vision under low-light conditions, while cone cells are responsible for color perception and high-resolution vision. The information generated by these two types of cells is integrated within the retina and then transmitted to the lateral geniculate nucleus (LGN) [37]. The LGN performs initial processing of visual information, such as detecting spatial orientation, motion direction, and speed. It has six layers, with layers 1, 4, and 6 processing input from one eye, and layers 2, 3, and 5 processing input from the other. The LGN fragments the image into patches and labels each fragment before transmitting them simultaneously to the primary visual cortex (V1).

The primary visual cortex (V1) and secondary visual cortex (V2) act as filters and feature extractors [38,39]. V1 detects basic visual features such as brightness, edges, and textures. Neurons in V1 respond selectively to specific orientations and spatial frequencies and can distinguish textures of varying thicknesses as well as different wavelengths of light. V2 contains neurons specialized in processing visual information at different scales and color features. It integrates the basic visual features from V1, enabling the rapid detection of important environmental information, and further builds depth perception before passing the information to V4.

The V4 and IT (inferotemporal) cortex regions are primarily responsible for pattern learning and memory [24,25,26]. Different areas within V4 respond differently to various patterns. Importantly, neurons in these regions do not respond only to one specific type of stimulus but rather show varying degrees and types of activation depending on the pattern. For example, neurons responsive to curved lines may also be activated to some extent by right-angled patterns. These responses are stored by neurons in the IT region. When similar stimuli enter the IT cortex again, it determines the type of input based on which neurons are activated and to what extent.

This is the biological process behind visual recognition in the human brain. Inspired by this mechanism, we propose a neural field model in this paper to address few-shot learning tasks.

## 3. Methodology

**Figure 2:**
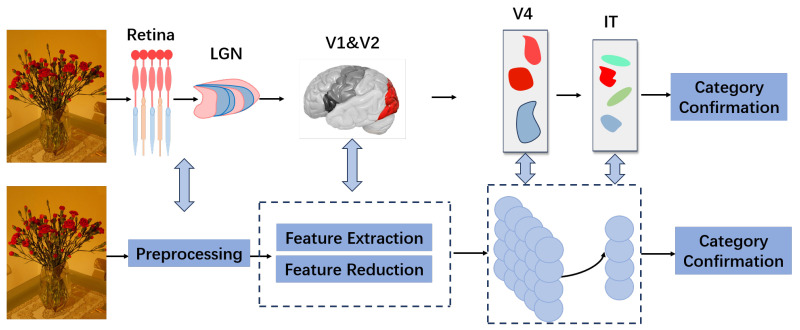
Overall structure of our model. It is divided into three functional modules: image preprocessing, feature extraction and dimensionality reduction, and the classifier. In the figure, these modules are mapped to the corresponding parts of the ventral stream structure in the brain that inspired them. Specifically, for the feature extraction and dimensionality reduction modules, we utilized the front-end feature extraction module of ViT. For the classifier module, we opted for a two-layer neural field.

Inspired by the ventral visual stream, in this section, we design a classifier for few-shot learning, as shown in Figure 2. We introduce a new neural field-based architecture for classification and provide some improvement methods to expand its applicability and computational efficiency.

### 3.1. Architecture of Two Fields

We employ two discrete neural fields to form a classifier. One of them is called the elementary field for feature representation, and the other one is called the high-level field for category representation. They are defined by the following equations:

(1) $$\tau_{u}{\dot{u}}_{i}\left(t\right)=\eta \left(\sum_{k=1}^{m}\omega_{u}\left(z_{i}-z_{k}\right)\phi \left(u_{k}\left(t\right)\right)\right)\;+e_{u,i}-u_{i}\left(t\right),\;i=1,2,\ldots ,m.$$

and

(2) $$\tau_{v}{\dot{v}}_{j}\left(t\right)=\eta \left(\sum_{k=1}^{m_{c}}\omega_{v}\left({\tilde{z}}_{j}-{\tilde{z}}_{k}\right)\phi \left(v_{k}\left(t\right)\right)\right)+\phi \left(\sum_{i=1}^{m}w_{j,i}\phi \left(u_{i}\left(t\right)\right)\right)+e_{v,j}-v_{j}\left(t\right),j=1,2,\ldots ,n.$$

$z_{i}\in Z,i=1,2,\ldots ,m$ are data samples for training. *n* is the number of classes. We suppose ${\tilde{z}}_{j}=\frac{1}{l_{j}}\sum_{i=1}^{l_{j}}{\tilde{z}}_{j_{i}}$ are class positions, i.e., the average positions of all their samples. The functions $u_{i}\left(t\right)$ and $v_{j}\left(t\right)$ describe the activation behaviors of the *i*th elementary neuron and the *j*th high-level neuron. $l_{j}$ is the number of samples in the *j*th class. $\tau_{f}$ and $\tau_{g}$ are positive evolution rates. ϕ(·) is a monotonically increasing, non-negative, and bounded activation function given by

$$\phi \left(u\right)=\left\{\begin{array}{cc} 1-\exp(-u), & u>0\\ 0, & u\leq 0 \end{array}\right..$$

The terms

$$\sum_{k=1}^{m}\omega_{u}\left(z_{i}-z_{k}\right)\phi \left(u_{k}\left(t\right)\right)$$

and

$$\sum_{k=1}^{m_{c}}\omega_{v}\left({\tilde{z}}_{j}-{\tilde{z}}_{k}\right)\phi \left(v_{k}\left(t\right)\right)$$

describe the lateral interactions of the elementary and high-level fields.

(3) $$\begin{array}{cc} \; & \omega_{u}\left(\tilde{z}\right)=A\exp\left(-\frac{1}{2}{∥\tilde{z}./\sigma_{u_{1}}∥}^{2}\right)-B\exp\left(-\frac{1}{2}{∥\tilde{z}./\sigma_{u_{2}}∥}^{2}\right), \end{array}$$

(4) $$\begin{array}{cc} \; & \omega_{v}\left(\tilde{z}\right)=A\exp\left(-\frac{1}{2}{∥\tilde{z}./\sigma_{v_{1}}∥}^{2}\right)-B\exp\left(-\frac{1}{2}{∥\tilde{z}./\sigma_{v_{2}}∥}^{2}\right), \end{array}$$

where

(5) $$A=\frac{1}{\sqrt{2\pi }\sigma_{u_{1}}},\;B=\frac{1}{\sqrt{2\pi }\sigma_{u_{2}}},$$

and

(6) $$\sigma_{u_{2}}=3\sigma_{u_{1}}.$$

We have A−B=1. Thus, $A=\frac{3}{2}$ and $B=\frac{1}{2}$, so we get that

(7) $$\begin{array}{cc} \; & \omega_{u}\left(\tilde{z}\right)=\frac{3}{2}\exp\left(-\frac{1}{2}{∥\tilde{z}./\sigma_{u_{1}}∥}^{2}\right)-\frac{1}{2}\exp\left(-\frac{1}{2}{∥\tilde{z}./\sigma_{u_{2}}∥}^{2}\right), \end{array}$$

(8) $$\begin{array}{cc} \; & \omega_{v}\left(\tilde{z}\right)=\frac{3}{2}\exp\left(-\frac{1}{2}{∥\tilde{z}./\sigma_{v_{1}}∥}^{2}\right)-\frac{1}{2}\exp\left(-\frac{1}{2}{∥\tilde{z}./\sigma_{v_{2}}∥}^{2}\right). \end{array}$$

By calculating zero points of the equations above, we get $∥\tilde{z}.∥=\frac{3\sqrt{ln3}}{2}\sigma_{u_{1}}$. η is the threshold function defined by

$$\eta \left(u\right)=\left\{\begin{array}{cc} 1-\exp(-u), & u>0\\ -1+\exp(u), & u\leq 0 \end{array}\right..$$

$\omega_{u}\left(\cdot \right)$ and $\omega_{v}\left(\cdot \right)$ are interaction kernels with a “Mexican hat” shape. $e_{u,i}$ and $e_{v,i}$ are binary input signals. $w_{j,i}$ is the connection weight between the *i*th neuron in the elementary field and the *j*th neuron in the high-level field.

### 3.2. Approximation of the Static Solution of Neural Fields

Since neural fields are dynamic, solving them consumes too much computation when the data dimensions are high. From observations in real-world classification tasks, we need to focus on the activation status of neurons in a neural field, but not on their specific values. Therefore, we do not have to solve the neural field equations with high accuracy. Additionally, when considering the classification problem of static data, the static solutions of the neural fields can be approximated by the convolution of the external input with an interaction kernel. Consequently, we can use the following two equations to approximate the solution of the original dynamic neural field equation for an external input $e_{f,k}$:

(9) $$u_{i}=\eta \left(\omega_{u}\left(z_{i}-z_{k}\right)\phi \left(e_{u,k}\right)\right),$$

and

(10) $$v_{j}=\eta \left(\sum_{k=1}^{m_{c}}\omega_{v}\left({\tilde{z}}_{j}-{\tilde{z}}_{k}\right)\phi \left(\sum_{i=1}^{m}x_{k,i}\phi \left(v_{i}\right)\right)\right)+\phi \left(\sum_{i=1}^{m}w_{j,i}\phi \left(u_{i}\right)\right).$$

When the data classes are distant, we can further suppose that the lateral interaction between their corresponding high-level neurons is weak and ignore the lateral interaction of the high-level field as follows:

(11) $$v_{j}=\phi \left(\sum_{i=1}^{m}w_{j,i}\phi \left(\eta \left(\omega_{u}\left(z_{i}-z_{k}\right)\phi \left(e_{u,k}\right)\right)\right)\right),j=1,2,\ldots ,n.$$

These are much simpler and require much less computation.

### 3.3. Feature Extraction and Preprocessing

Suppose that $L=\{Z_{1},Z_{2},\ldots ,Z_{m}\}$ is a support set, and $Z_{i},i=1,2,\ldots ,m$ are sample images. Images are highly unstructured data. Classification accuracy may be low if we input them into the model without feature extraction. To address this issue, we employ the feature extraction module from the front part of the Vision Transformer (ViT) to obtain image features as follows:

(12) $$x_{i}=F_{nn}\left(Z_{i}\right),i=1,2,\ldots ,m.$$

The extracted feature vector $x_{i}$ is high-dimensional and may contain information that is not useful for classification. This redundant information can reduce classification accuracy. Therefore, we employ some dimensionality reduction methods to preprocess them in the following way:

(13) $$z_{i}=dr_{nn}\left(x_{i}\right),i=1,2,\ldots ,m.$$

Let $Z=\{z_{1},z_{2},\ldots ,z_{m}\}$. We use a deep neural network pre-trained on the source task and keep it frozen during training on the target dataset.

### 3.4. Training and Prediction

#### 3.4.1. Training Phase

**Figure 3:**
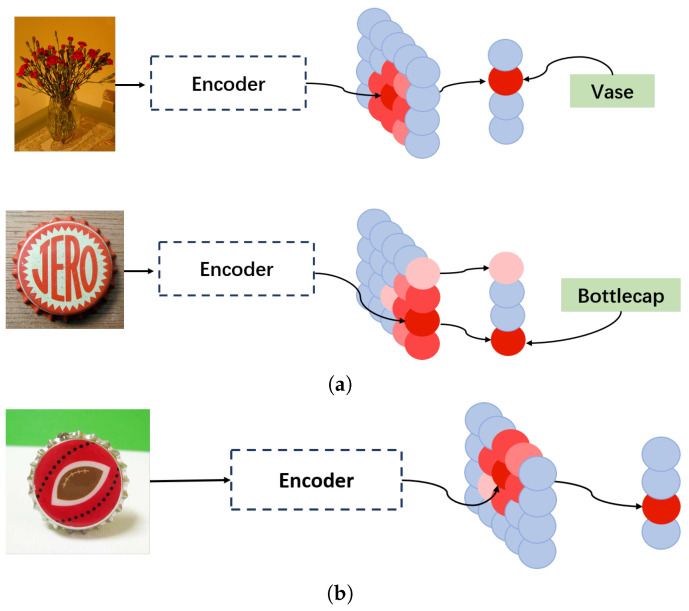
The training and prediction processes of the proposed model. (**a**) Training: Labeled samples from the training set are fed into the primary neural field, activating neurons in that region. Their labels correspond to specific neurons in the high-level neural field. A connection is then established between the two neural fields, indicating that the classifier module has successfully learned and memorized the features of that sample category. (**b**) Prediction: After a test sample is fed into the model, the model detects the region of neurons activated in the primary neural field and identifies the corresponding neuron in the high-level neural field. Subsequently, the model assigns the label associated with that neuron to the input test sample. Neurons shown in blue are inactive; the remaining neurons are colored in shades of red, with deeper red indicating stronger activation.

As we can see in Figure 3, the training process of our classifier is as follows: The training sample enters the primary neural field, which then establishes a connection with the high-level neural field based on the sample and its label. When a test sample is input into the primary neural field, the classifier determines the category by observing the activation state of neurons in the high-level neural field.

Based on the model structure we previously discussed, it is clear that the training process of the model is essentially about establishing a connection weight matrix W that links two layers of neural fields. When a labeled sample x is fed into the model, we assume that its feature vector and label activate the corresponding neurons in the primary and high-level neural fields. As a result, a connection is formed, and we set the connection weight to 1. Here, we have

$$w_{j,p}=\left\{\begin{array}{cc} 1 & {\hat{x}}_{p}={\hat{x}}^{\left(j\right)}\\ 0 & \mathrm{else} \end{array}\right.$$

#### 3.4.2. Adaptation of the Scale Parameter σ

Previously, we established the relationship between our scale parameter σ and the distance z, so we now focus on how to determine the value of z. Our few-shot learning tasks are specifically divided into two types: 5-way 1-shot and 5-way 5-shot. According to the definition of few-shot learning, we also categorize the selection of z into two corresponding cases. First, when there is only one sample per class, we compute the distance between samples from two classes and set z to be half of that distance. In contrast, when the support set contains more than one sample per class, we compute the distances between different samples within the same class and set z as the maximum of these distances. Subsequently, according to the relationship between z and σ described earlier, we can set the initial value of σ as

(14) $$\sigma_{u_{1}}=\frac{2}{3\sqrt{ln3}}z.$$

According to the 3-sigma rule, we set $\sigma_{max}$ and $\sigma_{min}$ as follows:

(15) $$\sigma_{max}=3\sigma_{u_{1}},\sigma_{min}=\frac{\sigma_{u_{1}}}{3}.$$

**Figure 4:**
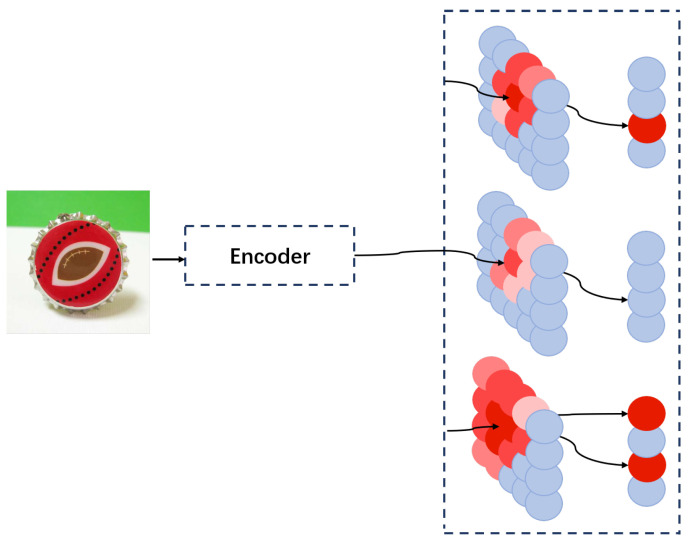
The three possible scenarios the model may encounter during prediction: (1) Only one neuron is activated. In this case, the model directly outputs the image category corresponding to the activated high-level neuron. (2) No neurons are activated. This indicates that the model’s perception range for high-level neurons is set too small. (3) Multiple neurons are activated. This suggests that the model’s perception range for high-level neurons is set too large. To address these issues, we incorporate a self-regulation process for the range parameters into the model, which significantly improves the accuracy of the experimental results. Neurons shown in blue are inactive; the remaining neurons are colored in shades of red, with deeper red indicating stronger activation.

During the prediction process, our model may encounter three scenarios: only one neuron in the high-level neural field is activated, multiple neurons are activated, or no neurons are activated, as shown in Figure 4.

The first scenario is the ideal case, where we can directly assign the category corresponding to the activated neuron to the sample. The other two scenarios indicate that there is an issue with the setting of our range parameter, which needs to be adjusted. Based on each situation, we apply the following adjustments, shown as Algorithm1. Before making any adjustments, we define a constant λ less than 1.

When no neurons are activated, we consider that the value of our range parameter is too small, resulting in no suitable neurons falling within the prediction range. Therefore, we enlarge the range parameter and perform prediction again using the updated value. We have:

(16) $$\sigma_{max}=\max\left\{\sigma_{max},\frac{1}{\lambda }\sigma_{u_{1}}\right\},\sigma min=\sigma_{u_{1}}.$$

Then, we get

(17) $$\sigma_{u_{1}}=\sigma_{max}-\lambda \left(\sigma_{max}-\sigma_{min}\right).$$

When multiple neurons are activated, we consider that the value of our range parameter is too large, allowing too many neurons to fall within the prediction range. Therefore, we reduce the range parameter and perform prediction again using the updated value. We have

(18) $$\sigma_{min}=\min\left\{\sigma_{min},\lambda \sigma_{u_{1}}\right\}.$$

According to the above two formulas, we have the following range parameter:

(19) $$\sigma_{f_{1}}=\sigma_{max}+\lambda \left(\sigma_{max}-\sigma_{min}\right).$$

**Algorithm 1** Scale Adaptation algorithm1:**Input:** $num,\sigma_{u_{1}},\lambda$2:**Output:** $\sigma_{u_{1}}$3:$\sigma_{max}=3\sigma_{u_{1}},\sigma_{min}=\frac{\sigma_{u_{1}}}{3}$4:**while** num≠1 **do**5:      **if** num=0 **then**6:         Let $\sigma_{max}=\max\left\{\sigma_{max},\frac{1}{\lambda }\sigma_{u_{1}}\right\}$7:         Calculate $\sigma_{u_{1}}=\sigma_{min}+\lambda \left(\sigma_{max}-\sigma_{min}\right)$8:      **else**9:         Let $\sigma_{min}=\min\left\{\sigma_{min},\lambda \sigma_{u_{1}}\right\}$10:          Calculate $\sigma_{u_{1}}=\sigma_{max}-\lambda \left(\sigma_{max}-\sigma_{min}\right)$11:     **end if**12:**end while**

## 4. Results

To demonstrate the proposed model, we compared the few-shot image classification accuracy of our model with that of other state-of-the-art models on three different datasets and studied the influence of different data dimensionality reduction methods, encoders, and feature extractors on the model through ablation experiments.

### 4.1. Datasets and Experimental Settings

In this experiment, we used the following datasets: Caltech-UCSD Birds-200-2011 (CUB200-2011) [40], CIFAR100 Few Shots (CIFAR-FS) [41], and miniImageNet [42], as shown in Table 1. They are summarized as follows:

• CUB200-2011: CUB200-2011 is a benchmark image dataset for fine-grained classification and recognition research. The dataset contains 11,788 images of birds across 200 subclasses.
• CIFAR-FS: The CIFAR-FS dataset (full name: the CIFAR100 Few Shots dataset) is derived from the CIFAR100 dataset. It contains 100 categories, with 600 images per category (60,000 images in total), each of size 32×32.
• miniImageNet: miniImageNet, derived from ImageNet, is intended for meta-learning and few-shot learning studies. It contains 60,000 84×84 color images across 100 categories, with 600 images per category.

Inspired by the biological structure shown in Figure 1, the overall structure of the proposed model is shown in Figure 2 and is divided into three modules: image preprocessing, feature extraction and dimensionality reduction, and the classifier. Given the limited number of labeled samples in few-shot learning, different methods of feature extraction can significantly affect the results. The classifier module, which is primarily responsible for learning and memorization tasks, plays a crucial role in few-shot learning tasks, as illustrated in Figure 3. Moreover, the adaptive component added to the module (see Figure 4) can further improve the experimental results.

**Table 1:** Number of classes and instances contained in the datasets used in this study.

	CUB200-2011	CIFAR-FS	miniImageNet
Instances	11,788	60,000	60,000
Classes	200	100	100

CUB-200-2011: a benchmark dataset for fine-grained image classification and recognition; CIFAR-FS: a few-shot learning dataset derived from CIFAR-100; miniImageNet: a subset of ImageNet commonly used for meta-learning and few-shot learning.

We used the same image processing method as [43]. Our ViT-L/16 model was pre-trained on the I21K dataset for ten epochs [4]. For dimensionality reduction, we employed LE (Laplacian Eigenmaps) [44] as $dr_{nn}$ to reduce the dimensionality to 4, with the number of neighbors in KNN for LE set to 99. On the miniImageNet and CIFAR-FS datasets, we used 80 classes for training and the remaining 20 for testing. On the CUB200-2011 dataset, we used 150 categories for training and the remaining 50 for testing.

### 4.2. Comparison with State-of-the-Art Models

We conducted 5-way 1-shot and 5-way 5-shot tasks for the proposed algorithm on CUB200-2011, CIFAR-FS, and miniImageNet. We compared the experimental results with those of EASY [16], ESPT [20], MetaQDA [17], PMF-BPA [21], SgVA-CLIP [15], P > M > F [18], and CAML [22]. The results are shown in Table 2, with the best results highlighted in bold and the second-best results underlined. For clarity, we use 5-1 and 5-5 in the tables to represent the 5-way 1-shot and 5-way 5-shot tasks, respectively. Additionally, we use CUB, CIFAR-FS, and MINI to represent CUB200-2011, CIFAR-FS, and miniImageNet.

The results in Table 2 show that overall, our method outperformed the comparison methods. We can see that on the CUB dataset, the performance of our method was slightly lower than that of the comparison methods. When we conducted the 5-way 1-shot task on this dataset, the result we obtained was 0.9242, which was second only to the 0.9580 achieved by PMF-BPA. However, in the 5-way 5-shot task, our model achieved a result of 0.9524, which was 1% lower than that of the other methods using the same backbone. Nevertheless, on the CIFAR-FS and miniImageNet datasets, the results obtained by our model were significantly higher than those of the comparison methods. On CIFAR-FS, the results we achieved for the 5-way 1-shot and 5-way 5-shot tasks were 0.9811 and 0.9846, which were much higher than those of the second-best model, PMF-BPA, which achieved results of 0.8710 and 0.9470. Lastly, on the miniImageNet dataset, our model obtained accuracies of 0.9887 and 0.9902. In the 5-way 1-shot task, the accuracy of the proposed model was at least 1% higher than that of the second-best model, while in the 5-way 5-shot task, it surpassed the SgVA-CLIP benchmark of 0.9872 by 0.3%. Although the accuracy of our model on the CUB dataset was not as high as that of the others, considering the overall performance on the three datasets, we believe that our proposed model is still superior to the comparison models.

### 4.3. Ablation Experiments

#### 4.3.1. Comparison with Different Distance Metrics

The distance metric may influence accuracy. We investigated the accuracy of the LE method with the Euclidean distance, correlation distance, cosine distance, and Minkowski distance. We present the results in Table 3. The best results are highlighted in bold, and the second-best ones are underlined.

The results obtained with the cosine distance on these three datasets were 0.9242, 0.9524, 0.9811, 0.9846, 0.9887, and 0.9902. In comparison, the second-best results obtained using the other distance metrics for dimensionality reduction were 0.9125, 0.9494, 0.9785, 0.9816, 0.9873, and 0.9899. Therefore, we can conclude that the cosine distance achieved the highest accuracy in each task on every dataset.

#### 4.3.2. Comparison with Different Feature Extractors and Encoders

To demonstrate the superiority of the classifier in our model, we compared it with KNN and SVM on the CUB200-2011, CIFAR-FS, and miniImageNet datasets, using the same feature extractor and dimensionality reduction method. Additionally, to eliminate the influence of the feature extractor, we employed ViT and ResNet18 as the feature extractors. The experimental results are presented in Table 4, with the best results highlighted in bold, and the second-best results underlined.

As shown in Table 4, in both the 5-way 1-shot and 5-way 5-shot tasks on the CUB, CIFAR-FS, and miniImageNet datasets, the accuracy of our proposed model was at least 1% higher than that of KNN when using the same feature extractor for the same task. Similarly, Table 4 shows that the accuracy of SVM was consistently lower than that of our model across all three datasets. Notably, in the 5-way 1-shot tasks, the accuracy of SVM was nearly 20% lower than that of our model on various datasets. At the same time, we can see from the results in Table 4 that different feature extractors impacted the experimental results. We noticed that for the same few-shot learning tasks on the same dataset, using ViT as the feature extractor yielded results that were 8–30% higher than those obtained using ResNet18 as the feature extractor.

## 5. Discussion

In this study, inspired by the physiological structure of the ventral visual stream, we designed a classifier module capable of few-shot learning based on the neural field equation. We coupled this classifier module with the feature extraction part of the Vision Transformer (ViT) to compensate for ViT’s shortcomings in few-shot learning. Figure 1 illustrates the biological structure of the ventral visual stream, which mainly includes the retina, lateral geniculate nucleus, and cortical areas V1, V2, V4, and IT. Given the close association of this brain region with few-shot learning capabilities, we attempted to design a classifier module suitable for few-shot learning tasks based on this structure. The datasets summarized in Table 1 are internationally recognized benchmark datasets for evaluating few-shot learning methods. This motivated our biologically inspired design, aiming to provide a novel solution to few-shot learning challenges.

As shown in Figure 2, the few-shot learning model we designed is divided into three main parts: image preprocessing, feature extraction and dimensionality reduction, and the classifier. Since the number of labeled samples in few-shot learning is limited, the feature extraction module plays a crucial role in the entire model structure. After obtaining image features from the feature extraction module, the primary neural field of the classifier module memorizes the labeled samples and their categories, thereby improving the accuracy of the classifier’s predictions for the categories of test samples. The comparative results in Table 2 demonstrate that our method consistently outperforms baseline models across multiple datasets, confirming the benefits of integrating biological principles with modern deep architectures. From the overall model architecture shown in Figure 2, it can be seen that feature extraction and dimensionality reduction are key components that significantly impact the experimental results. Therefore, Table 3 primarily demonstrates how different distance parameters used for dimensionality reduction under the same conditions affect the experimental outcomes.

Figure 3a shows the training process of the classifier, which combines supervised learning and meta-learning. Supervised learning ensures that the model can accurately classify examples, while meta-learning helps the model quickly adapt to new tasks. This training process is particularly effective for few-shot learning because it enables the model to generalize well from a small number of examples. The results in Table 2 indicate that this training process is highly effective in enhancing the model’s performance in few-shot learning tasks. Figure 3b illustrates the prediction process of our model. This process involves invoking the features learned during training and matching the features of test samples with the learned features for prediction. The model’s ability to extract features from a small number of samples and generalize is crucial for few-shot learning, and our classifier module is designed specifically to achieve this capability.

Figure 4 shows the adjustments made to the range-perception parameters in the model during the experimental process based on the prediction results. When the model fails to detect activated neurons, it indicates that the range-perception parameters are set too small; conversely, when the model detects multiple activated neurons, it suggests that the range-perception parameters are set too large. Both situations can significantly impact the experimental results. Therefore, we added a self-regulation module to the model to adjust the range-perception parameters. By introducing this module, our experimental results were further improved and stabilized. To better demonstrate the stability of the experimental results, the results shown in Table 2 are the averages of 600 experiments, which effectively illustrate that our model can achieve high precision and stability in few-shot learning tasks.

Although our model achieves high precision on real-world datasets, its application results on medical images are still not satisfactory. Therefore, in future work, we will further improve and optimize this model.

## Figures and Tables

**Table 2:** Accuracy of the proposed model compared with that of EASY, ESPT, MetaQDA, PMF-BPA, SgVA-CLIP, and CAML on CUB200-2011, CIFAR-FS, and miniImageNet. Best results are in bold, second-best are underlined.

Method (Backbone)	CUB(5-1)	CUB(5-5)	CIFAR-FS(5-1)	CIFAR-FS(5-5)	MINI(5-1)	MINI(5-5)
EASY (ResNet12) [16]	0.7856	0.9193	0.7620	0.8900	0.7175	0.8715
ESPT (ResNet12) [20]	0.8545	0.9402	\	\	0.6836	0.8411
ProtoNet (ResNet18) [45]	0.7188	0.8742	0.7220	0.8350	0.5416	0.7368
ProtoNet (ViT) [45]	0.8700	0.9710	0.5770	0.8100	0.8530	0.9600
MetaQDA (ViT) [17]	0.8830	**0.9740**	0.6040	0.8320	0.8820	0.9740
CAML (ViT) [22]	0.9180	0.9710	0.7080	0.8476	0.9620	0.9860
DeepEMD (ViT) [19]	\	\	0.8280	0.9310	0.9050	0.9720
PMF-BPA (ViT) [21]	**0.9580**	0.9712	0.8710	0.9470	0.9520	0.9870
P >M > F (ViT) [18]	0.9230	0.9700	0.8430	0.9220	0.9530	0.9840
FSViT (ViT) [23]	\	\	0.8370	0.9360	0.9590	0.9850
SgVA-CLIP (ViT) [15]	\	\	\	\	0.9795	0.9872
Ours (ViT)	0.9242	0.9524	**0.9811**	**0.9846**	**0.9887**	**0.9902**

The methods compared include EASY (Ensemble Augmented-Shot-Y-Shaped Learning), ESPT (Episodic Spatial Pretext Task), ProtoNet (Prototypical Network), MetaQDA (Meta-learning generalization of Quadratic Discriminant Analysis), CAML (Context-Aware Meta-Learning), DeepEMD (Differentiable Earth Mover’s Distance), PMF-BPA (Pairwise-Mutual-Feature Balanced-Pairwise-Affinities transform), P >M > F (Pre-training, Meta-training, and Fine-tuning), FSViT (Multi-Scale Feature Sets in Vision Transformer), SgVA-CLIP (Semantic-guided Visual Adapting for vision–language pre-trained models such as CLIP), ViT (Vision Transformer), and ResNet12 (a 12-layer residual network with four residual stages and a final global average-pooling layer).

**Table 3:** Effects of different distance metrics on model accuracy on CUB200-2011, CIFAR-FS, and miniImageNet. The best results are highlighted in bold, and the second-best results are underlined.

Metric	CUB(5-1)	CUB(5-5)	CIFAR-FS(5-1)	CIFAR-FS(5-5)	MINI(5-1)	MINI(5-5)
Euclidean	0.6831	0.7409	0.7178	0.7861	0.7878	0.8243
Cosine	**0.9242**	**0.9524**	**0.9811**	**0.9846**	**0.9887**	**0.9902**
Correlation	0.9125	0.9494	0.9785	0.9816	0.9873	0.9899
Minkowski	0.8839	0.9192	0.9517	0.9596	0.9772	0.9825

Distances used in this study include the Euclidean distance, which measures the straight-line distance between two points; the cosine distance, defined as the cosine of the angle between two vectors; the correlation distance, computed as the Pearson correlation coefficient; and the Minkowski distance, a generalized distance metric with parameter p.

**Table 4:** Accuracy of different feature extractors and classifiers. The best results are highlighted in bold, and the second-best results are underlined.

Encoder	Classifer	CUB(5-1)	CUB(5-5)	CIFAR-FS(5-1)	CIFAR-FS(5-5)	MINI(5-1)	MINI(5-5)
ViT	KNN	0.9099	0.9302	0.9678	0.9726	0.9720	0.9786
	SVM	0.7462	0.9445	0.7670	0.9747	0.7914	0.9833
	OURS	**0.9242**	**0.9524**	**0.9811**	**0.9846**	**0.9887**	**0.9902**
Resnet18	KNN	0.8300	0.8632	0.7768	0.8272	0.6495	0.7201
	SVM	0.6420	0.8670	0.5825	0.8185	0.4956	0.7150
	OURS	**0.8435**	**0.8738**	**0.7912**	**0.8380**	**0.6743**	**0.7374**

The classifiers used in this study include KNN, a non-parametric classifier that assigns the majority label among the k closest training samples based on the chosen distance metric; SVM, a maximum-margin classifier that finds an optimal hyperplane to separate classes, optionally using kernel functions for non-linear decision boundaries; ViT, the Vision Transformer; and ResNet18, a standard 18-layer residual network containing an initial 7 × 7 convolution, four residual blocks, and a global average-pooling layer.

## Data Availability

The original data presented in the study are openly available in GitHub at https://github.com/Sue214313/FSL, accessed on 16 July 2025.

## Abbreviations

The following abbreviations are used in this manuscript:

ViT	Vision Transformer
LGN	Lateral geniculate nucleus
V1	Primary visual cortex
V2	Secondary visual cortex
IT	Inferior temporal cortex
